# microRNA‐106b derived from endothelial cell–secreted extracellular vesicles prevents skin wound healing by inhibiting JMJD3 and RIPK3

**DOI:** 10.1111/jcmm.16037

**Published:** 2021-03-18

**Authors:** Lin Qi, Yufeng Lu, Zhaolin Wang, Guiyun Zhang

**Affiliations:** ^1^ Department of Dermatology The Second Hospital of Jilin University Changchun China; ^2^ Department of Thoracic Surgery China‐Japan Union Hospital of Jilin University Changchun China; ^3^ Department of Orthopaedics China‐Japan Union Hospital of Jilin University Changchun China

**Keywords:** extracellular vesicles, fibroblasts, keratinocytes, microRNA‐106b, skin wound healing

## Abstract

Intriguingly, microRNAs (miRs) transferred as cargo in extracellular vesicles (EVs) can modulate wound healing through their regulation of fibroblast functions. In this study, we investigated the effects of miR‐106b transfer via EVs derived from human umbilical vein endothelial cells (HUVECs) on skin wound healing. Dual‐luciferase reporter gene assay identified that miR‐106b could target and inhibit JMJD3. RT‐qPCR analysis showed EVs isolated from HUVECs had enriched expression of miR‐106b. LL29 fibroblast cells and HaCaT keratinocytes were co‐cultured with HUVEC‐derived EVs, in which miR‐106b had been up‐regulated or down‐regulated by its mimic or inhibitor. The co‐culture with HUVEC‐derived EVs increased miR‐106b expression, and reduced the viability and adhesion of LL29 and HaCaT cells, whereas the inhibition of miR‐106b in HUVEC‐derived EVs enhanced the viability and adhesion of LL29 and HaCaT cells through up‐regulation of JMJD3. Next, we showed that JMJD3 overexpression enhanced LL29 and HaCaT cell viability and adhesion through elevating RIPK3, which induced the phosphorylation of AKT during the wound‐healing process. We next developed a mouse skin wound model to investigate the actions of miR‐106b in vivo after 14 days. The delivery of miR‐106b *via* HUVEC‐derived EVs delayed wound healing through suppression of collagen I content and angiogenesis, but had no effects on pro‐inflammatory cytokines. In conclusion, miR‐106b from HUVEC‐derived EVs inhibits JMJD3 and RIPK3, leading to the inhibition of skin wound healing, thus constituting a new therapeutic target.

## 1 INTRODUCTION

Research on tissue repair is assuming great importance in recent decades, particularly in chronic diseases such as diabetic ulcer, yet the comprehensive mechanisms and pre‐conditions for tissue repair have not been defined. Extracellular vesicles (EVs) are an important vehicle for transferring cargo for cell‐to‐cell communication, being involved not only in physiological processes, but also in cancer and metabolic diseases.^1^, ^2^ In the present context, EVs have been shown to modulate wound healing.^3^, ^4^ EVs carry various molecules for cell communication, including non‐coding RNAs, metabolites, proteins, lipids and microRNAs (miRs).^5^ Among these cargoes, miRs such as miR‐23b and miR‐31 have been implicated in skin wound healing.^6^, ^7^ More importantly, miRs conveyed by EVs participate in the modulation of wound healing through regulating angiogenesis and fibroblast functions.^8^ In particular, EVs derived from endothelial cells suppress skin wound healing through regulating the autophagy of fibroblasts.^9^ Investigating miRs derived from EVs can improve our understanding of regulators of human skin homeostasis in fibroblasts and keratinocytes,^10^ which promises to deliver key information about crucial signalling molecules that participate in the process of skin wound healing.

miRs have been shown to inhibit the differentiation of fibroblasts and therefore inhibit wound healing.^11^ miR‐106b is a well‐known miR in the cancer research field, having been shown to inhibit cell cycle progression in some cancers.^12^, ^13^ A recent report has suggested that miR‐106b derived from endothelial cell–secreted EVs can inhibit skin wound healing.^9^ Therefore, our first aim in this study was to investigate the role of miR‐106b conveyed by EVs in skin wound healing using a mouse model.

Jumonji domain–containing protein‐3 (JMJD3), also known as KDM6B, is a histone H3K27 demethylase involved in HOX gene regulation and development.^14^, ^15^ As such, JMJD3 can elevate gene expression by enhancing demethylation of the promoter region. Interestingly, JMJD3 has been shown to promote skin wound healing in keratinocytes through interacting with an inflammatory transcription factor NF‐κB.^16^ However, the interaction between JMJD3 and miR‐106b remains unknown. Therefore, our second aim was to determine whether miR‐106b from EVs interacts with JMJD3 in skin wound healing.

Receptor‐interacting serine/threonine‐protein kinase 3 (RIPK3) is part of the tumour necrosis factor (TNF) receptor I signalling complex, which activates NF‐κB.^17^, ^18^ A previous study has shown that RIPK3 can promote skin wound healing,^19^ and other work shows that RIPK3 can activate AKT^20^ to promote skin wound healing.^21^ In the light of the above findings, we explored the possibility that RIPK3 could activate AKT to promote skin wound healing via an axis downstream of miR‐106b and JMJD3.

## 2 MATERIALS AND METHODS

### 2.1 Ethics statement

All experiments involved animals were performed in accordance with the recommendations in the Guidelines for the Care and Use of Laboratory Animals issued by National Institutes of Health. Animal protocols were approved by the Animal Ethics Committee of The Second Hospital of Jilin University (Approval Number: 201908003).

### 2.2 Cell culture

Human umbilical vein endothelial cells (HUVECs) and human dermal fibroblast LL29 from Institute of Dermatology, Chinese Academy of Medical Sciences (Nanjing, China), and human immortalized keratinocyte cell line HaCaT from Kunming Cell Bank of Type Culture Collection of Chinese Academy of Sciences (Kunming, China) were maintained in DMEM (Invitrogen, Waltham, MA), 10% foetal bovine serum (FBS) and 100 U/mL penicillin/streptomycin (Invitrogen) at 37°C with 5% CO_2_. Medium was changed every 3‐4 days. LL29 cells were passaged upon attaining 70%‐90% confluence and observed for adherence and the presence of spindle‐shaped structure under an inverted microscope. HaCaT cells were proved to be free‐floating and circular‐shaped. Cells at passages 3‐6 were used for experiments.

### 2.3 Extraction and identification of HUVEC‐derived EVs

An EV extraction kit was used to collect and purify EVs from the culture supernatant of HUVECs (SBI, Palo Alto, CA). The supernatant was centrifuged at 300 *g* for 10 minutes, at 2000 *g* for 30 minutes, and then at 10 000 *g* for 30 minutes. The supernatant was concentrated by passage through an Amicon Ultra‐15 Centrifugal Filter (100 kDa; Millipore, Burlington, MA). Ultrafiltration liquid and EV separation reagent were mixed at a ratio of 4:1 and incubated at 4°C for more than 12 hours. The mixture was then centrifuged at 1500 *g* for 30 minutes. The EV pellet was resuspended in 200 μL phosphate buffer saline (PBS), and protein content was measured using a bicinchoninic acid (BCA) kit (Beyotime Biotechnology, Shanghai, China). The isolated EV pellets were stored at −80°C for future experiments.

The size and morphological characteristics of EVs were examined by transmission electron microscopy (TEM). An EV suspension was mixed with an equal volume of 4% paraformaldehyde and placed on a formvar carbon‐coated EM grid. Images were acquired using a TEM (Hitachi, Tokyo, Japan), and the EV surface marker proteins CD63 (ab59479; Abcam, Cambridge, UK) and CD81 (ab79559, Abcam) were quantified by Western blot analysis using selective antibodies. EVs were labelled with carboxyfluorescein succinimidyl ester (CFSE, 10 μmol/L; Thermo Fisher Scientific, Waltham, MA) at 37°C for 30 minutes. The labelled EVs were then washed with PBS and centrifuged at 100 000 *g* for 1 hours to remove excess dye.^22^ For tracking of EVs in vitro, CFSE‐labelled EVs were co‐cultured with LL29 or HaCaT cells in culture medium for 4 hours and observed with Olympus BX41 microscope equipped with a charge‐coupled detector device (Magnafire, Vancouver, BC, Canada).

### 2.4 Cell transfection

When LL29 or HaCaT cells reached 90% density and were in logarithmic growth phase, the cells were trypsinized and dispersed to 2.5 × 10^4^ cells/mL suspension. The cells were seeded at a density of 2 mL/well into a 6‐well plate. When the confluence reached 30%, cells were infected with lentivirus expressing overexpression (oe)‐negative control (NC), lentivirus expressing short hairpin RNA (sh)‐NC, lentivirus expressing shRNA against JMJD3 (sh‐JMJD3), lentivirus expressing RIPK3 (oe‐RIPK3) or lentivirus expressing shRNA against JMJD3 (sh‐RIPK3) for 2‐3 d. The sequences were constructed by GenePharma (Shanghai, China) with shRNAs shown in Table 1. Lentivirus (2 × 10^6^ TU), 5 μg polybrene, 1 mL of serum‐free and antibacterial drug‐containing medium were mixed for infection. Forty‐eight hours after infection, 1 μg/mL of puromycin was added to each well to select stably infected cells. After successful lentiviral infection, cells were cultured until reaching 70%‐80% confluence. Cells were then transiently transfected with miR‐106b mimic and miR‐106b inhibitor using Lipofectamine^TM^ 2000 Reagent (Invitrogen), following the manufacturer's instructions. Cells were collected 48 hours after transfection for subsequent experiments. In addition, cells infected with lentivirus expressing oe‐NC or oe‐RIPK3 were co‐cultured with EVs isolated from HUVECs infected with lentivirus expressing miR‐NC or miR‐106b. All experiments were performed in triplicate.

**Table 1:** Plasmid sequences for cell transfection

shRNA	Sequence
sh‐JMJD3	5′‐CCGGCCTGTTCGTTACAAGTGAGAACTCGAGTTCTCACTTGTAACGAACAGGTTTTTG‐3′
sh‐JMJD3‐1	5′‐TGCACTCAGGATTCGCATAACCTTCAAGAGAGGTTATGCGAATCCTGAGTGCTTTTTTC‐3′
sh‐RIPK3	5′‐CCAGCACUCUCGUAAUGAUTT‐3′
sh‐RIPK3‐1	5′‐AACTGGAGGCAGGGAAAACT‐3′

Note

shRNA; sh‐JMJD3, shRNA against JMJD3; sh‐RIPK3, shRNA against RIPK3; JMJD3, jumonji domain–containing protein‐3; RIPK3, receptor‐interacting serine/threonine‐protein kinase 3.

### 2.5 Dual‐luciferase reporter gene assay

A full‐length JMJD3 3′ untranslated region (UTR) sequence was obtained from gene sequence database and inserted downstream in the firefly luciferase gene driven by the SV40 promoter in the pEZX‐MT01 vector to generate a recombinant vector PEZX‐MT01‐JMJD3 3′UTR‐wild‐type (WT) and PEZX‐MT01‐JMJD3 3′UTR‐mutant type (MUT). HEK293T cells (American Type Culture Collection, Manassas, VA, USA) were co‐transfected with PEZX‐MT01‐JMJD3 3′UTR‐WT or PEZX‐MT01‐JMJD3 3′UTR‐MUT and NC mimic or miR‐106b mimic using Lipofectamine 2000 Reagent. Cells were cultured for 48 hours, and the luciferase activity was measured using a Dual‐Glo® Luciferase Assay System (Promega, Madison, WI) and an EnVision 2102 Multilabel Plate Reader (Perkin Elmer Inc, Waltham, MA). Renilla luciferase activity was used as an internal reference. The relative luciferase activity was the ratio between Firefly and Renilla luciferase activity.

### 2.6 Animal treatment

Male C57BL/6 mice (8‐10 weeks old, n = 40) were provided by Experimental Animal Center of The Second Hospital of Jilin University. All operations were performed under sodium pentobarbital anaesthesia (pentobarbital sodium inj., USP 50 mg/mL, Akorn, Lake Forest, IL lot 080103F).

Mice with a skin wound were injected subcutaneously with PBS containing EVs at concentration of 25 mg/100 mL at four different sites daily. The mice were injected with EVs derived from untreated HUVECs (EVs) or were co‐injected with EVs derived from cells treated with miR‐NC (EV‐miR‐NC) or EVs derived from cells treated with miR‐106b (EV‐miR‐106b) and lentivirus expressing oe‐NC or oe‐RIPK3. Lentiviral vectors and reagents were purchased from GenePharma.

### 2.7 Skin wound animal model

Mice were anaesthetized with 3% sodium pentobarbital. Their backs were shaved and sterilized with 10% povidone‐iodine, and a wound measuring 1 × 1 cm was made on the skin. The wound closure area was photographed and measured daily during dressing changes until the 14th day. At days 1, 4, 7, 10 and 14, the wound surface area was measured. After euthanasia, the full‐thickness skin samples including the wound and the epithelial margin were collected. Full‐thickness skin samples from intact mice were used as controls. One half of the skin samples were fixed and stored in 10% formalin for histological analysis, and the other half were frozen in liquid nitrogen and stored at −80°C for qPCR analysis.

### 2.8 Measurement of the rate of wound healing

Wound area on the back of the mice was photographed and tracked daily until day 14, and the captured images were analysed by ImageJ (Rayne Rasband software 1.48q, National Institutes of Health, Bethesda, MD). Wound‐healing ratio was calculated according to the following formula: wound‐healing area (%) = (area n)/area 0] × 100, where area 0 is the initial wound area, and area n is the area on day n after injury.

### 2.9 Haematoxylin and eosin (HE) staining and Masson's Trichrome Staining

Injured skin tissue was harvested, fixed with 10% formalin, dehydrated and embedded in paraffin. Tissues were sliced into 5‐μm‐thick sections and transferred to glass slides. The histological staining assays were performed in accordance with HE Staining Kit instructions (Sigma‐Aldrich, St. Louis, MO) and Masson's Trichrome Staining Kit (Bogoo Biotechnology, Shanghai, China). Tissue sections were permeabilized with 0.1% Triton X‐100 (Sigma) for 10 minutes and incubated with a 3% hydrogen peroxide in methanol for 20 minutes. After blocking with goat serum for 1 hour at room temperature, sections were incubated with antibodies against smooth muscle actin α (α‐SMA, myofibroblast marker) for 2 hours. Subsequently, sections were incubated with a biotin‐labelled secondary antibody for 30 minutes and then incubated with streptavidin peroxidase for 30 minutes at 37°C. Nuclei were stained with haematoxylin, and collagen was stained with Masson's Trichromatic dye. Morphological changes in the skin tissues were visualized under an optical microscope and imaged.

### 2.10 Cell viability determined by 3‐(4, 5‐dimethylthiazol‐2‐yl)‐2, 5‐diphenyltetrazolium bromide (MTT) assay

LL29 or HaCaT cells (0.2 mL per well) were added to a 96‐well culture plate and incubated with MTT solution. Optical density (OD) was read at 450 nm at 24, 48 and 72 hours, and the cell adhesion was measured at 72 hours. Experiments were repeated three times and averaged.

### 2.11 Chromatin immunoprecipitation (ChIP)‐quantitative polymerase chain reaction (qPCR)

Cells were collected and cross‐linked with 1% formaldehyde for 10 minutes at room temperature. After sonication, soluble chromatin fragments were incubated with protein A (#16‐661, Millipore) or protein G (#16‐662, Millipore) antibodies. Immunoprecipitated complexes were then eluted. DNA was extracted and purified by QIAquick PCR Purification Kit (Qiagen, Hilden, Germany). The ChIP DNA, normalized to input DNA, was determined by qPCR using primers (Table 2). The results from three independent experiments were averaged.

**Table 2:** Primer sequences for ChIP‐qPCR

Gene	Human sequence
JMJD3	F: 5′‐GCCTGACCTCTACCACCTAC‐3′
	R: 5′‐CTTCTGTGGCTGCTGATGAC‐3′
RIPK3	F: 5′‐GCGTTGGCTCTCACTAAAGG‐3′
	R: 5′‐CTGCTAGAAGCCATGGTGTG‐3′

Note

ChIP‐qPCR, chromatin immunoprecipitation‐quantitative polymerase chain reaction; F, forward; R, reverse; JMJD3, jumonji domain–containing protein‐3; RIPK3, receptor‐interacting serine/threonine‐protein kinase 3.

### 2.12 mRNA expression determined by reverse transcription (RT)‐qPCR

Total cellular RNA was extracted by TRIzol™ Reagent (Invitrogen). miR‐106b was converted into cDNA using miR‐X miRNA First‐Strand Synthesis Kit (Takara, Kusatsu, Japan). For mRNA quantification, cDNA was synthesized using the PrimeScript™ RT Kit (Takara). Real‐time qPCR was conducted with SYBR Premix Ex Taq II (Takara) in an ABI 7500 Real‐Time PCR System (Applied Biosystems, Foster City, CA) to determine RNA levels of miR‐106b (normalized to U6), JMJD3 and RIPK3 (normalized to β‐actin) (Table 3). The RNA level was calculated by the 2^−ΔΔCT^ method. Experiments were repeated three times independently to obtain an average.

**Table 3:** Primer sequences for RT‐qPCR

Gene	Primer sequence
miR‐106b (human)	5′‐TAAAGTGCTGACAGTGCAGAT‐3′
miR‐106b (mouse)	5′‐AGCCGTCAAGAGCAATAACGAA‐3′,
JMJD3 (human)	F: 5′‐TGGACTACTTGACGGGTTCC‐3′
	R: 5′‐TGGTACTGATAGGCGGTGAG‐3′
JMJD3 (mouse)	F: 5′‐CCCCCATTTCAGCTGACTAA‐3′
	R: 5′‐CTGGACCAAGGGGTGTGTT‐3′
RIPK3 (human)	F: 5′‐ATGTCGTGCGTCAAGTTATGG‐3′
	R: 5′‐CGTAGCCCCACTTCCTATGTTG‐3′
RIPK3 (mouse)	F: 5′GGCACCCTAGCGTACTTGG‐3′
	R: 5′‐GCTGTAGACATCACTCGCTTT‐3′
U6	F: 5′‐GGAACGATACAGAGAAGATTAGC‐3′
	R: 5′‐TGGAACGCTTCACGAATTTGCG‐3′
β‐actin	F: 5′‐TGTCCACCTTCCAGCAGATGT‐3′
	R: 5′‐AGCTCAGTAACAGTCCGCCTAGA‐3′

Note

RT‐qPCR, reverse transcription‐quantitative polymerase chain reaction; F, forward; R, reverse; miR‐106b, microRNA‐106b; JMJD3, jumonji domain–containing protein‐3; RIPK3, receptor‐interacting serine/threonine‐protein kinase 3.

### 2.13 Protein expression determined by Western blot analysis

Cell lysates were obtained using a radio‐immunoprecipitation assay lysis buffer (Beyotime Institute of Biotechnology) containing a protease and a phosphatase inhibitor (Thermo Scientific). Protein concentration was measured using a BCA method. Protein was then electrophoresed on a polyacrylamide gel (5% spacer gel and 12% separation gel) and transferred to a polyvinylidene fluoride membrane (Millipore). The membrane was blocked at room temperature with a tri‐buffered saline (TBST) buffer containing 5% bovine serum albumin (BSA) on a shaker for 1 hour. Primary antibody was prepared with 5% BSA. The membrane was incubated with primary antibodies against CD63 (1:1000, ab59479, Abcam), CD81 (1:1000, ab109201, Abcam), calnexin (1:1000, ab10286, Abcam), JMJD3 (1:1000, ab169197, Abcam), RIPK3 (1:1000, ab62344, Abcam), AKT (1:250, ab8805, Abcam), p‐AKT (1:500, ab38449, Abcam), collagen I (1:1000, ab34710, Abcam), keratinocyte chemokine (KC) (SRP4251, murine interleukin [IL]‐8 homolog, Sigma‐Aldrich), IL‐1β (1:500, ab9722, Abcam), tumour necrosis factor‐α (TNF‐α) (1:3500, ab9739, Abcam), vascular endothelial growth factor (VEGF) (1:500, sc‐507, Santa Cruz), transforming growth factor (TGF)‐β1 (1:1500, ab92486, Abcam), β‐actin (1:1000, ab5694, Abcam) and glyceraldehyde‐3‐phosphate dehydrogenase (1:1000, ab8245, Abcam) at 4°C overnight. Then, the membrane was incubated with secondary goat anti‐rabbit antibody (1:2,000, ab6721, Abcam) or goat anti‐mouse antibody (1:2,000, ab205719, Abcam) at 4°C for 4‐6 hours. The membrane was then washed with TBST (3 × 15 minutes). Chemiluminescence reagents (Yanhui Biotechnology Co., Ltd., Shanghai, China) were mixed and added to the film for development. Grey values were obtained in all immunoblotted bands. Experiments were repeated three times independently to obtain an average.

### 2.14 Statistical analysis

All data were analysed by SPSS 21.0 statistical software (IBM Corp., Armonk, NY). Data were expressed as mean ± standard deviation. Data comparison between two groups was performed by unpaired *t* test. Data at different time‐points (OD values) were analysed by two‐way analysis of variance (ANOVA). Wound‐healing rate at different time‐points was analysed by repeated‐measures ANOVA with the Bonferroni post hoc test. Differences were considered significant when *P* < .05.

## 3 RESULTS

### 3.1 miR‐106b from HUVEC‐derived EVs targets JMJD3 and inhibits cell viability and adhesion

**FIGURE 1:**
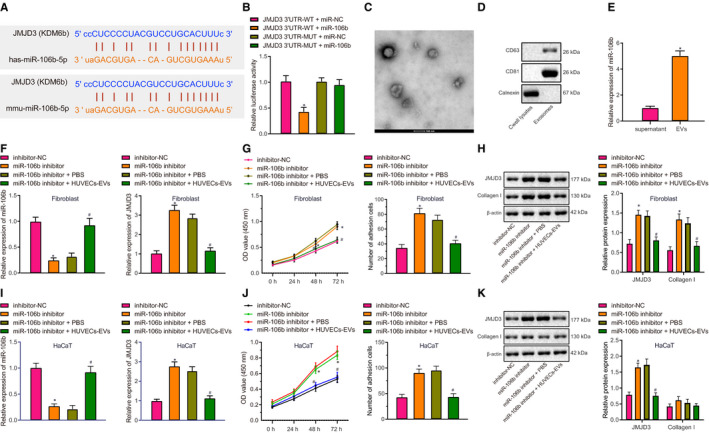
miR‐106b from HUVEC‐derived EVs targets JMJD3 and inhibits adhesion and viability of fibroblasts and keratinocytes. A, Binding site of miR‐106b to JMJD3 (KDM6B) 3′UTR in humans and mice predicted by the Starbase database. B, Binding relationship between miR‐106b and JMJD3 identified by dual‐luciferase reporter gene assay. ^*^ *P* < .05 vs JMJD3 3′UTR‐WT + miR‐NC group. C, Representative electronic microscopic images showing HUVEC‐derived EVs (scale bar: 100 nm). D, Expression of EV surface marker protein CD63 and CD81 and endoplasmic reticulum marker, calnexin, as measured by Western blot assay. E, The miR‐106b expression in EVs determined by RT‐qPCR. ^*^ *P* < .05 vs supernatant. F, The miR‐106b and JMJD3 expression in LL29 cells determined by RT‐qPCR. G, Viability and adhesion of LL29 cells determined by MTT assay. H, The protein expression of JMJD3 and collagen I in LL29 cells measured by Western blot assay. I, The expression of miR‐106b and JMJD3 in HaCaT cells determined by RT‐qPCR. J, Viability and adhesion of HaCaT cells assessed by MTT assay. K, JMJD3 and collagen I protein expressions in HaCaT cells measured by Western blot assay. In panels F‐K, ^*^ *P* < .05 vs inhibitor‐NC group; ^#^ *P* < .05 vs miR‐106b inhibitor + PBS group. Data were expressed as mean ± standard deviation, and comparison between two groups was performed by unpaired *t* test. Data at different time‐points (OD values) were analysed by two‐way ANOVA

Prediction from the Starbase database revealed that the JMJD3 3′UTR was complementary to the miR‐106b seed sequence (Figure 1A). We next used dual‐luciferase reporter gene assay to verify this binding relationship. miR‐106b and JMJD3 3′UTR‐WT co‐transfection led to lower luciferase activity, whereas miR‐106b and JMJD3 3′UTR‐MUT co‐transfection did not have much effect on luciferase activity (Figure 1B). Hence, miR‐106b could directly target JMJD3.

Particles isolated from HUVEC supernatant were cup‐shaped or sac‐like and had an average diameter of 145 ± 3.2 nm, demonstrating the successful isolation of secreted EVs (Figure 1C). HUVEC‐derived EVs were positive for CD63 and CD81, but negative for calnexin (Figure 1D). By RT‐qPCR determination, miR‐106b was highly expressed in EVs derived from HUVECs (Figure 1E).

The process of skin wound healing often involves the adhesion and proliferation of fibroblasts and keratinocytes, as well as changes in the expression of collagen I.^23^ We conducted miR‐106b loss‐of‐function experiments in fibroblasts and keratinocytes, which showed that miR‐106b inhibitor transfection appreciably reduced miR‐106b expression in LL29 cells (Figure 1F). miR‐106b inhibitor led to an increase in JMJD3 expression, adhesion and viability of LL29 cells, and collagen I expression (Figure 1F‐H). However, the co‐culture with HUVEC‐derived EVs normalized the effects of miR‐106b inhibitor on the adhesion and viability of LL29 cells as well as JMJD3 and collagen I expression. Consistently, miR‐106b inhibitor transfection notably reduced miR‐106b expression in HaCaT cells (Figure 1I). miR‐106b loss of function resulted in remarkably increased JMJD3 expression and adhesion and viability of HaCaT cells, but had no effect on collagen I expression in HaCaT cells (Figure 1I‐K). The co‐culture with HUVEC‐derived EVs also neutralized the effects of miR‐106b inhibitor on adhesion and viability of HaCaT cells as well as JMJD3 expression. These data indicated that miR‐106b, derived from HUVEC‐derived EVs, targeted JMJD3 and reduced adhesion and viability of fibroblasts and keratinocytes, whereby preventing the healing of skin wounds.

### 3.2 JMJD3 promotes skin wound healing through up‐regulation of RIPK3 in human fibroblasts and keratinocytes

**FIGURE 2:**
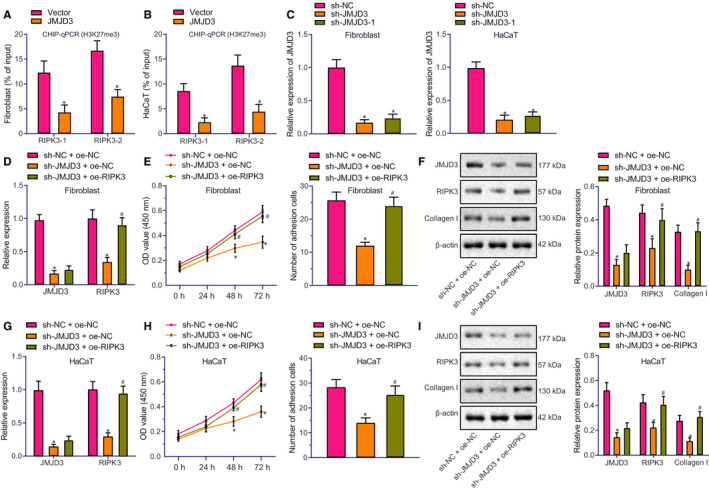
JMJD3 promotes adhesion and viability of human fibroblasts and keratinocytes through up‐regulation of RIPK3. A and B, H3K27me3 expression in human LL29 cells (A) and HaCaT cells (B) after JMJD3 overexpression. ^*^ *P* < .05 vsvector group. C, JMJD3 expression after infection with lentivirus expressing sh‐JMJD3 in human LL29 cells and HaCaT cells. ^*^ *P* < .05 vs sh‐NC group. D, JMJD3 and RIPK3 expression in human LL29 cells determined by RT‐qPCR. E, Cell viability and adhesion of LL29 cells determined by MTT assay. F, Protein expression of JMJD3, RIPK3 and collagen I in LL29 cells measured in human LL29 cells by Western blot assay. G, JMJD3 and RIPK3 expression in human HaCaT cells determined by RT‐qPCR. H, Cell viability and adhesion of HaCaT cells determined by MTT assay. I, Protein expression of JMJD3, RIPK3 and collagen I in HaCaT cells measured by Western blot assay. In panels D‐I, ^*^ *P* < .05 vs sh‐NC + oe‐NC group; ^#^ *P* < .05 vs sh‐JMJD3 + oe‐NC group. Data were expressed as mean ± standard deviation, and comparison between two groups was performed by unpaired *t* test. Data at different time‐points (OD values) were analysed by two‐way ANOVA with post hoc correction

We conducted ChIP‐qPCR assays to determine the enrichment of H3K27me3 in the RIPK3 promoter region. JMJD3 overexpression reduced H3K27me3 in the RIPK3 promoter region in both LL29 cells and HaCaT cells (Figure 2A,B), indicating that JMJD3 catalysed the demethylation of H3K27me3 in the RIPK3 promoter region, leading to RIPK3 up‐regulation.

sh‐JMJD3 and sh‐JMJD3‐1 were designed to knockdown JMJD3 in human LL29 cells and HaCaT cells. As shown in Figure 2C. sh‐JMJD3 exhibited a higher knockdown efficiency than did sh‐JMJD3‐1, and therefore, sh‐JMJD3 was selected for subsequent experiments. As expected, infection with lentivirus expressing sh‐JMJD3 led to decreased JMJD3 expression, whereas the infection of lentivirus expressing oe‐RIPK3 increased RIPK3 expression in LL29 cells (Figure 2D). JMJD3 knockdown reduced adhesion and viability of LL29 cells, and lowered collagen I expression in LL29 cells (Figure 2D‐F), but these changes induced by JMJD3 knockdown were normalized by RIPK3 overexpression. JMJD3 knockdown decreased adhesion and viability of HaCaT cells and collagen I expression (Figure 2G‐I), but these effects were likewise normalized by RIPK3 overexpression. The above results indicated that JMJD3 promoted the viability and adhesion of fibroblasts and keratinocytes by elevating the expression of RIPK3, thereby facilitating the skin wound healing.

### 3.3 RIPK3 phosphorylates AKT in human fibroblasts and keratinocytes to promote skin wound healing

**FIGURE 3:**
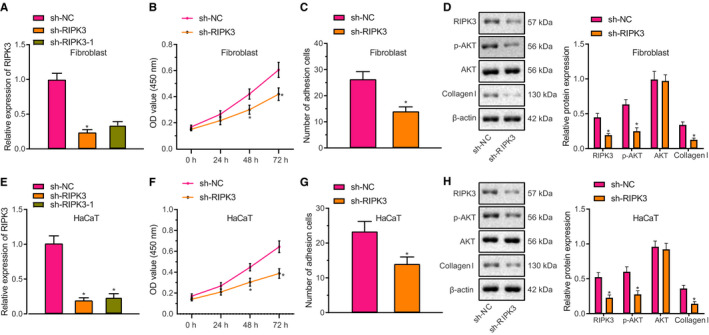
RIPK3 promotes adhesion and viability of human fibroblasts and keratinocytes through phosphorylation of AKT. A, Expression of RIPK3 in human LL29 cells after infection with lentivirus expressing sh‐RIPK3 determined by RT‐qPCR. B and C, Viability (B) and adhesion (C) of LL29 cells assessed by MTT assay. D, Protein expression of RIPK3, phosphorylated AKT, AKT and collagen I in LL29 cells measured by Western blot assay. E, Expression of RIPK3 in human HaCaT cells after infection with lentivirus expressing sh‐RIPK3 determined by RT‐qPCR. F and G, Viability (F) and adhesion (G) of HaCaT cells determined by MTT assay. H, Protein expression of RIPK3, phosphorylated AKT, AKT and collagen I in HaCaT cells measured by Western blot assay. ^*^ *P* < .05 vs sh‐NC group. Data were expressed as mean ± standard deviation, and comparison between two groups was performed by unpaired *t* test. Data at different time‐points (OD values) were analysed by two‐way ANOVA with post hoc correction

sh‐RIPK3 and sh‐RIPK3‐1 vectors were designed to knock down RIPK3 in human LL29 cells and HaCaT cells. sh‐RIPK3 exhibited higher knockdown efficiency than that of sh‐RIPK3‐1 and was therefore selected for use in further experiments (Figure 3A). RIPK3 knockdown decreased adhesion and viability of LL29 cells, and reduced collagen I expression in LL29 cells (Figure 3B‐D). RIPK3 knockdown reduced the phosphorylation level of AKT in LL29 cells, whereas AKT expression was unchanged by this treatment (Figure 3D). In HaCaT cells, RIPK3 knockdown also inhibited cell adhesion and viability, and down‐regulated collagen I expression and the phosphorylation level of AKT (Figure 3E‐H). Hence, RIPK3 can promote the viability and adhesion of fibroblasts and keratinocytes by phosphorylation of AKT, thereby facilitating the skin wound healing.

### 3.4 miR‐106b from HUVEC‐derived EVs targets inhibition of JMJD3 in human fibroblasts and keratinocytes to inhibit skin wound healing

**FIGURE 4:**
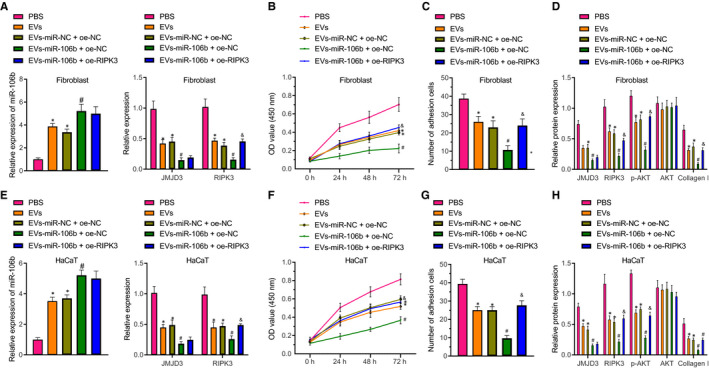
EVs containing miR‐106b impede adhesion and viability of human fibroblasts and keratinocytes through down‐regulation of RIPK3. A, The expression of miR‐106b, JMJD3 and RIPK3 in LL29 cells determined by RT‐qPCR. B and C, Viability (B) and adhesion (C) of LL29 cells assessed by MTT assay. D, Expression of JMJD3, RIPK3, AKT, phosphorylated AKT and collagen I proteins in LL29 cells measured by Western blot assay. E, The expression of miR‐106b, JMJD3 and RIPK3 in HaCaT cells determined by RT‐qPCR. F and G, Viability (F) and adhesion (G) of HaCaT cells assessed by MTT assay. H, Expression of JMJD3, RIPK3, AKT, phosphorylated AKT and collagen I proteins in HaCaT cells measured by Western blot assay. ^*^ *P* < .05 vs the PBS group; ^#^ *P* < .05 vs the EV‐miR‐NC + oe‐NC group; ^&^ *P* < .05 vs the EV‐miR‐106b + oe‐NC group. Data were expressed as mean ± standard deviation, and comparison between two groups was performed by unpaired *t* test. Data at different time‐points (OD values) were analysed by two‐way ANOVA with post hoc correction

The LL29 cells and HaCaT cells infected with lentivirus expressing oe‐NC or oe‐RIPK3 or untransfected LL29 cells and HaCaT cells were co‐cultured with PBS, with EVs derived from untreated HUVECs, or with HUVECs transfected with miR‐106b or miR‐NC (namely EVs, EV‐miR‐106b or EV‐miR‐NC). Compared with PBS treatment, the expression of miR‐106b was significantly increased, but cell adhesion and viability and collagen I, JMJD3, RIPK3 and phosphorylated AKT expression were decreased, but AKT expression was unaffected in LL29 cells after EV treatment. Similarly, EV‐miR‐106b treatment elevated miR‐106b expression but reduced cell adhesion and viability, whereas collagen I, JMJD3, RIPK3 and phosphorylated AKT expression were decreased together with unchanged AKT expression in LL29 cells, which was neutralized by overexpressing RIPK3 (Figure 4A‐D). Furthermore, similar results were observed in HaCaT cells undergoing the same treatments (Figure 4E‐H). These results show that miR‐106b carried by HUVEC‐derived EVs inhibited RIPK3 and phosphorylation of AKT through targeted inhibition of JMJD3, leading to inhibition of skin wound healing.

### 3.5 miR‐106b restrains mouse skin wound healing through targeted inhibition of JMJD3

**FIGURE 5:**
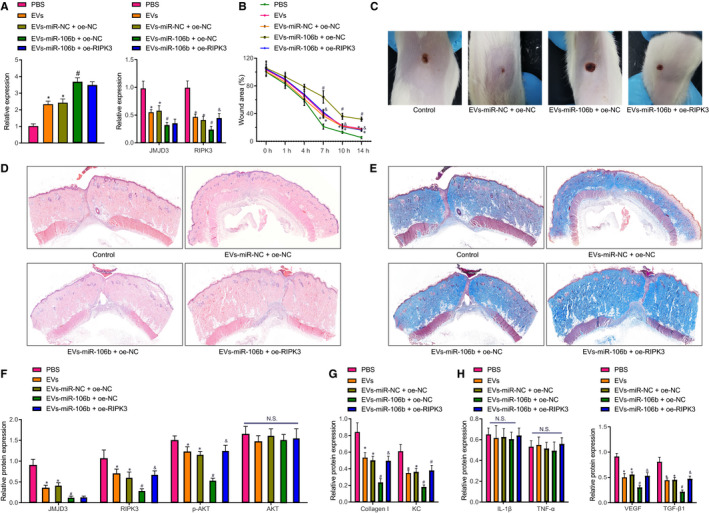
miR‐106b inhibits RIPK3 and phosphorylation of AKT through inhibiting JMJD3 to restrain mouse skin wound healing. A, The expression of miR‐106b, JMJD3 and RIPK3 in mice determined by RT‐qPCR. B, Skin wound‐healing area on days 1, 4, 7 and 14. C, Photographs of wound healing in mice on day 7 post‐injury. D, Epithelial tissue morphology and angiogenesis observed by H&E staining on day 7. E, Collagen content determined by Masson staining. F, Expression of JMJD3, RIPK3, phosphorylated AKT and AKT normalized to β‐actin measured by Western blot assay on day 7. G, Expression of collagen I and chemokine KC normalized to β‐actin in keratinocytes measured by Western blot assay on day 7. H, Expression of pro‐inflammatory proteins (IL‐1β and TNF‐α) and angiogenesis proteins (VEGF and TGF‐β1) normalized to β‐actin measured by Western blot assay on day 7. ^*^ *P* < .05 vs the PBS group; ^#^ *P* < .05 vs the EV‐miR‐NC + oe‐NC group; ^&^ *P* < .05 vs the EV‐miR‐106b + oe‐NC group; NS, no significant difference. Data were expressed as mean ± standard deviation, and comparison between two groups was performed by unpaired *t* test (n = 10). Wound‐healing area at different time‐points was analysed by repeated‐measures ANOVA with the Bonferroni post hoc test

Finally, the effects of miR‐106b from HUVEC‐derived EVs on wound healing were tested in the in vivo wound model. Treatment with EVs or EV‐miR‐106b, especially EV‐miR‐106b treatment, increased miR‐106b expression but decreased JMJD3 and RIPK3 expression in C57BL/6 mice with the in vivo wound model. Moreover, decreased RIPK3 expression caused by EV‐miR‐106b was negated by overexpressing RIPK3 (Figure 5A). Wound‐healing area decreased with increasing time, as shown in Figure 5B,C. On day 7, EVs or EV‐miR‐106b, especially EV‐miR‐106b treatment, reduced wound‐healing area in vivo, but this effect was normalized by RIPK3 overexpression. On day 7, HE staining results showed obvious angiogenesis and epithelium regeneration in mice injected with EVs, EV‐miR‐NC + oe‐NC or EV‐miR‐106b + oe‐RIPK3 (Figure 5D). However, no epithelium regeneration was observed in the mice injected with EV‐miR‐106b + oe‐NC. In addition, total collagen content was lower in mice injected with EV‐miR‐106b + oe‐NC than in control mice or mice injected with EV‐miR‐NC + oe‐NC or EV‐miR‐106b + oe‐RIPK3 (Figure 5E). Moreover, EV‐miR‐106b reduced protein expression of JMJD3, RIPK3 and phosphorylation of AKT but caused little change in AKT protein expression (Figure 5F). The overexpression of RIPK3 had no effect on the expression of JMJD3, but enhanced the phosphorylation of AKT and RIPK3 expression. These results show that miR‐106b, derived from HUVEC‐derived EVs, targeted JMJD3 in vivo and inhibited RIPK3 and phosphorylation of AKT, whereby inhibiting skin wound healing.

We further examined the changes in collagen I and KC. EV‐miR‐106b diminished the contents of collagen I and KC (Figure 5G), but up‐regulation of RIPK3 neutralized the effects of EV‐miR‐106b on these markers. The results suggest that miR‐106b transferred from HUVEC‐derived EVs inhibited the progression of chemotaxis in fibroblasts and keratinocytes during skin wound healing.

As inflammation may occur during skin wound healing, we determined pro‐inflammatory cytokines IL‐1β and TNF‐α in the mouse model. Compared with PBS treatment, there were no significant differences regarding IL‐1β and TNF‐α in mice after treatment with EVs, EV‐miR‐NC + oe‐NC, EV‐miR‐106b + oe‐NC or EV‐miR‐106b + oe‐RIPK3 (Figure 5H).

As angiogenesis is involved in wound healing, we determined the expression of angiogenesis proteins VEGF and TGF‐β1 in the mouse model. Compared with PBS treatment, treatment with EVs or EV‐miR‐NC + oe‐NC caused down‐regulation of VEGF and TGF‐β1. Compared with treatment with EV‐miR‐NC + oe‐NC, treatment with EV‐miR‐106b + oe‐NC also diminished the expression of VEGF and TGF‐β1 (Figure 5H). The reductions in VEGF and TGF‐β1 induced by EV‐miR‐106b were rescued by lentivirus‐mediated RIPK3 overexpression. Hence, miR‐106b transferred from HUVEC‐derived EVs might inhibit the angiogenesis to slow the skin wound healing.

## 4 DISCUSSION

There are a few important findings in this study. First, we show enrichment of miR‐106b in HUVEC‐derived EVs, which inhibited adhesion and viability of fibroblasts and keratinocytes, the two main cell types that are essential in skin wound healing. Meanwhile, we found that miR‐106b targeted JMJD3, which in turn up‐regulated the expression of RIPK3. Additionally, JMJD3 promoted skin wound healing in a mouse model through up‐regulation of RIPK3, which enhanced the phosphorylation of AKT. Finally, we found that miR‐106b overexpression in vitro and in vivo repressed skin wound healing through up‐regulating RIPK3. miR‐106b overexpression also reduced chemotaxis of fibroblasts and angiogenesis, but not affect the release of pro‐inflammatory cytokines. These results suggest that miR‐106b from HUVEC‐derived EVs had a negative impact on skin wound healing, whereby antagonism of miR‐106b may be a new therapeutic target meriting further investigation.

EVs are crucially involved in cell communications during diverse physiological and pathological processes,^1^, ^2^ including the process of wound healing.^3^, ^4^ EVs carry many molecules for cellular communication, including miRs.^5^ Our study demonstrated that miR‐106b delivered by EVs inhibited skin wound healing. This agrees with the finding of a previous study showing the role of miR‐106b in wound healing.^9^ Other studies also showed that miR‐23b and miR‐31 participated in skin wound healing.^6^, ^7^ The mechanism of wound healing is complex, probably involving multiple miRs and many downstream mediators. Although by no means completely understood, previous work shows that miRs may alter immune response or Wnt9b signalling during wound healing.^24^, ^25^

To the best of our knowledge, this is the first demonstration that miR‐106b can target and inhibit JMJD3, which is a histone H3K27 demethylase.^14^ We also found that JMJD3 was involved in skin wound healing. The importance of this finding lies in part in the discovery that JMJD3 could enhance keratinocyte wound healing,^16^ and also insofar as JMJD3 up‐regulated RIPK3 by virtue of its demethylase characteristics. RIPK3 is part of the TNF receptor I signalling complex, which activates NF‐κB transcription factor.^17^, ^18^ Indeed, we find that RIPK3 promotes skin wound healing through the activation of AKT, which is consistent with a previous study.^20^ AKT is a well‐known signalling molecule in the vasculature, which has multiple effects in endothelial and smooth muscle cells.^26^, ^27^ AKT may promote skin healing by promoting angiogenesis starting from the endothelium through its regulation of the phosphatidylinositol‐3 kinase signalling pathway,^27^ which may present an important topic for further studies.

We studied the effects of EV‐encapsulated miR‐106b on the functions of fibroblasts and keratinocytes because these cell types are both important in skin wound healing. In fact, these two cell types interact with each other during skin wound healing *via* delivery of EVs.^10^ Our study revealed the inhibitory effects of EV‐encapsulated miR‐106b on the adhesion and viability of fibroblasts and keratinocytes. On the other hand, we elaborated the role of EV‐encapsulated miR‐106b in angiogenesis, which is also critical in wound healing.^8^ The present study results also showed that that expression of VEGF and TGF‐β1, which are two important mediators of angiogenesis,^28^, ^29^ was both reduced by treatment with EV‐encapsulated miR‐106b, further suggesting that miR‐106b may inhibit skin wound healing through the inhibition of the angiogenesis process. However, we did not find any corresponding alteration of pro‐inflammatory cytokines, suggesting that inflammation per se may not be affected by EV‐encapsulated miR‐106b in this skin wound‐healing model.

**FIGURE 6:**
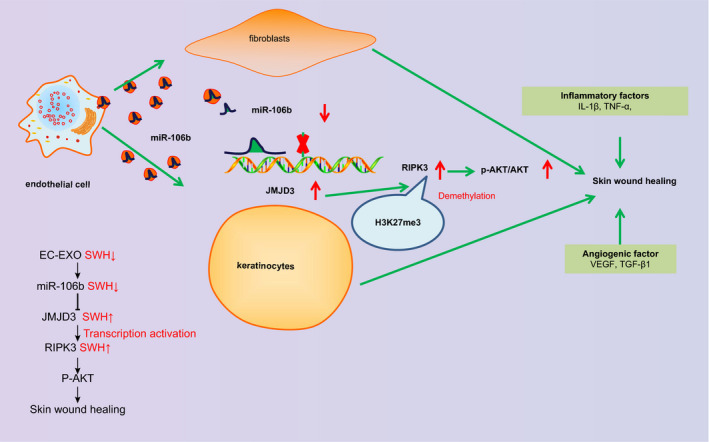
Inhibition of miR‐106b in HUVEC‐derived EVs up‐regulates JMJD3 and RIPK3, contributing to skin wound healing

In conclusion, the inhibition of miR‐106b mediated by HUVEC‐secreted EVs enhances skin wound healing in a mouse model through up‐regulating JMJD3 and RIPK3 (Figure 6), suggesting that EV‐encapsulated miR‐106b present a promising strategy for promoting skin wound healing. These results may have implications for disorders of wound healing, such as in diabetic ulcer or wound infection. Although we examined the critical functions of fibroblast and keratinocytes in skin wound healing, we have not yet established their interaction in this process. Therefore, determining their interaction in relation to enhanced wound healing presents an interesting future research direction.

## CONFLICT OF INTEREST

The authors confirm that there are no conflicts of interest.

## AUTHOR CONTRIBUTIONS

**Lin Qi:** Conceptualization (lead); Methodology (equal); Writing‐original draft (equal); Writing – review and editing (equal). **Yufeng Lu:** Data curation (lead); Investigation (lead); Writing – review and editing (equal). **Zhaolin Wang:** Formal analysis (lead); Writing – review and editing (equal). **Guiyun Zhang:** Methodology (equal); Writing‐original draft (equal); Writing – review and editing (equal).

## Data Availability

All the data supporting the findings of this study are available within the article.
